# Development and validation of an intelligent weight verification management system for neonatal ready-to-use parenteral nutrition infusions

**DOI:** 10.1371/journal.pone.0351932

**Published:** 2026-08-18

**Authors:** Xiaolin Tang, Chunhui Zhu, Dan Cheng, Jin Zhu, Lijuan Qiu, Xiaoxia Han, Chuanhua Feng, Mengying Chen

**Affiliations:** 1 Department of Pharmacy, Children’s Hospital of Jiangxi Province, Nanchang, Jiangxi, P.R.China; 2 Department of Pharmacy, The First Affiliated Hospital, Jiangxi Medical College, Nanchang University, Nanchang, Jiangxi, P.R.China; Assistance Publique - Hôpitaux de Marseille, FRANCE

## Abstract

**Objective:**

To develop an intelligent weight verification management system for the quality of ready-to-use neonatal parenteral nutrition (PN) infusions and validate its reliability and application efficacy, aiming to enhance the quality and safety of these infusions.

**Methods:**

An intelligent weight verification approach was established through drug density determination, maintenance of information parameters, automatic calculation of theoretical weight by the hospital information system (HIS), and actual measurement using a high-precision electronic scale. A total of 350 bags of neonatal PN orders prepared in the Pharmacy Intravenous Admixture Services (PIVAS) of our hospital from January to June 2024 were randomly selected. Seven compounding technicians prepared 50 bags each, and two designated pharmacists conducted verification using the residual liquid method and the weighing method, respectively. The error rate between the theoretical and actual weighing values was computed, statistical analysis of the pertinent data was conducted, and the viability of the weighing method was assessed from four perspectives: precision, trend controllability, operator stability, and verification efficiency.

**Results:**

The relative standard deviation (RSD) of error rates was < 1%, indicating excellent precision. The average error rate was 1.92%, with 97.43% of error rates within the predefined threshold (−1% to 5%) and 90.9% within the 3σ range. The error rate trends for different volumes aligned with variations in filling quantities, demonstrating controllable overall error trends. The interquartile range (IQR) variation in error rates among operators at different time points was < 30%, reflecting good operational stability. The weighing method achieved significantly higher verification efficiency (15.4 minutes per bag) than the residual liquid method (28.6 minutes per bag, P < 0.05) and identified an additional 6 unqualified infusions.

**Conclusion:**

The weight verification system demonstrates validated reliability in improving quality assurance and operational efficiency for ready-to-use neonatal PN infusions in PIVAS settings, warranting clinical implementation.

## 1. Introduction

Parenteral nutrition (PN) refers to the complete or partial intravenous provision of calories, fluids, proteins, carbohydrates, fats, vitamins, and minerals to meet the body’s metabolic and growth requirements when patients cannot tolerate or can only partially tolerate enteral feeding [1]. PN has been widely adopted in neonatal medicine, with research demonstrating that early and proactive PN therapy can effectively improve the survival success rate of critically ill neonates, particularly preterm infants. It has now become a fundamental treatment modality in the neonatal intensive care unit (NICU). Due to the immature development of the neonatal metabolic system and the relatively delicate balance of body fluids and electrolytes, neonates have stringent requirements for the compositional ratios and concentrations of individual components in PN. Therefore, ensuring the quality of ready-to-use neonatal PN infusions is of paramount and self-evident importance [2].

It is well established that PN formulations involve complex prescription components, with each order comprising 4–14 medications. Neonatal PN poses unique challenges due to non-integer vial usage (nearly all doses require partial vials) and stringent dosing precision requirements (sub-milliliter accuracy), rendering quality verification of ready-to-use PN infusions particularly demanding [3]. Current neonatal PN verification methods, including dual-operator verification and residual volume measurement (also termed the residual-liquid method), remain prone to subjective variability. Notably, in high-volume Pharmacy Intravenous Admixture Service (PIVAS) workflows, these methods demonstrate increased risks of inspection oversights [4]. To enhance error detection efficacy, our PIVAS team conducted a systematic literature review and identified weight verification as a promising management protocol (hereafter termed the weighing method) [5]. While existing studies primarily address adult PN verification [6–9], standardized protocols for neonatal small-volume, high-precision applications remain underdeveloped. In response, our PIVAS team developed a verification management model for ready-to-use neonatal PN infusions based on the weighing method. The methodological details and validation outcomes are presented below.

## 2. Materials and methods

### 2.1. Instruments

Calibrated high-precision electronic balance (±0.1 g, Shanghai Hengliang Weighing Equipment Co., Ltd.), densitometer (room temperature 20°C, Alcon Medical), and standard syringes (The specifications include 1 mL, 5 mL and 10 mL, Jiangxi Sanxin Medical Technology Co., Ltd.).

### 2.2. Drugs

The drugs included those commonly used in neonatal PN orders at our hospital: 0.9% Sodium Chloride Injection, 5% Glucose Injection, 50% Glucose Injection, Pediatric Compound Amino Acid Injection (18AA-I), Multi-Oil Fat Emulsion Injection, 10% Sodium Chloride Injection, 10% Potassium Chloride Injection, Magnesium Sulfate Injection (25%), Calcium Gluconate Injection, Fructose Sodium Diphosphate Injection, Pediatric Multivitamin Injection (13), and Multiple Trace Elements Injection, etc. These were classified by volume into large-volume injections (e.g., 0.9% sodium chloride, 5% glucose) and small-volume injections (e.g., 50% glucose, 10% sodium chloride).

### 2.3. Samples

A total of 350 bags of ready-to-use neonatal PN infusions prepared in our hospital’s PIVAS from January to June 2024 were randomly selected. Seven compounding technicians participated in the admixture process, with each preparing 50 bags. Two designated pharmacists independently verified the admixtures using the residual-liquid method and the weighing method, respectively. Data were systematically collected to validate the feasibility of the weighing method.

### 2.4. Definition of the residual liquid method operation

The Residual Liquid Method, which served as the study’s control, is the standard quality recheck method for neonatal parenteral nutrition infusions in the Pharmacy Intravenous Admixture Services (PIVAS) of our hospital. The precise operational procedure is as follows: each component drug’s residual liquid is retained after parenteral nutrition is prepared; the volume of the residual liquid is measured independently using a graduated scale, and the deviation rate between the actual residual liquid and the theoretical residual liquid is calculated. Deviation rates that fall within ±5% are considered qualified. This approach, which is based on manual measurement and calculation, is appropriate for the preliminary verification of small-volume prepared infusions, but it is susceptible to factors such as residual liquid wall adhesion and human reading errors.

### 2.5. Density determination

#### 2.5.1. Densitometer measurement.

For large-volume drugs such as 0.9% sodium chloride, 5% glucose, fat emulsion, and amino acids, three batches of each drug were selected, with six samples drawn from each batch. Drug densities were directly measured using a densitometer at room temperature of 20°C (Table 1). The relative standard deviation (RSD) values of drug densities in the table were all ≤ 1%, indicating excellent measurement precision.

**Table 1:** Densities of Large-Volume Drugs (g/mL).

Drugs	Batch	Sample1	Sample2	Sample3	Sample4	Sample5	Sample6	Mean	RSD
0.9% Sodium Chloride	1	1.006	1.005	1.004	1.007	1.008	1.006	1.006	0.13%
	2	1.005	1.008	1.007	1.006	1.008	1.007		
	3	1.009	1.005	1.006	1.005	1.006	1.005		
5% Glucose	1	1.018	1.017	1.017	1.017	1.016	1.016	0.001	0.12%
	2	1.016	1.015	1.017	1.018	1.017	1.016		
	3	1.017	1.018	1.019	1.015	1.016	1.015		
Fat Emulsion	1	0.978	0.988	0.989	0.990	0.976	0.989	0.004	0.48%
	2	0.986	0.979	0.988	0.989	0.991	0.977		
	3	0.985	0.86	0.979	0.992	0.988	0.989		
Fructose Sodium Diphosphate	1	1.005	1.002	1.001	1.001	1.003	1.001	1.002	0.18%
	2	1.003	1.000	1.001	1.006	1.002	1.002		
	3	1.004	1.001	0.999	1.002	1.002	1.003		
Amino Acids 18AA	1	1.022	1.021	1.023	1.019	1.020	1.018	1.020	0.25%
	2	1.024	1.019	1.018	1.017	1.018	1.025		
	3	1.016	1.020	1.021	1.023	1.024	1.021		

#### 2.5.2. Mass-volume conversion.

For small-volume drugs such as 50% glucose, 10% sodium chloride, 10% potassium chloride, calcium gluconate, and multiple vitamins 13.In order to prevent drug waste, the drug density was determined using the mass-volume conversion formula: ρ = m/V (where ρ is the drug density in g/mL, m is the drug mass in g, and V is the drug volume in mL.Three batches of each drug were selected, with six measurements performed per batch. Using 1 mL, 5 mL, and 10 mL syringes, 1 mL, 3 mL, 5 mL, 7 mL, 9 mL, and 10 mL of the drug solution were drawn and weighed separately, and the drug density was calculated using the mass-volume conversion formula (Table 2). The RSD values of drug densities in the table were all ≤ 1%, indicating excellent precision.

**Table 2:** Densities of Small-Volume Drugs (g/mL).

Drugs	Batch	1mL	3mL	5mL	7mL	9mL	10mL	Mean	RSD
10% Sodium Chloride	1	1.064	1.067	1.065	1.059	1.058	1.069	1.064	0.21%
	2	1.063	1.059	1.065	1.061	1.059	1.069		
	3	1.060	1.066	1.067	1.059	1.069	1.066		
50% Glucose	1	1.164	1.167	1.159	1.163	1.165	1.168	1.165	0.40%
	2	1.160	1.162	1.166	1.163	1.164	1.165		
	3	1.158	1.169	1.164	1.164	1.165	1.164		
10% Potassium Chloride,	1	1.058	1.060	1.061	1.059	1.057	1.062	1.059	0.24%
	2	1.060	1.061	1.059	1.056	1.058	1.057		
	3	1,057	1.058	1.060	1.065	1.055	1.060		
Calcium Gluconate	1	1.075	1.080	1.056	1.067	1.061	1.066	1.064	0.63%
	2	1.071	1.069	1.059	1.060	1.063	1.062		
	3	1.059	1.053	1.060	1.068	1.066	1.063		
Multiple Vitamins 13	1	1.019	1.018	1.025	1.031	1.032	1.020	1.025	0.49%
	2	1.019	1.028	1.026	1.024	1.029	1.031		
	3	1.021	1.019	1.026	1.023	1.032	1.030		

### 2.6. Measurement of empty PN infusion container weight

Due to the small liquid volume, our hospital used 5% glucose injection in various specifications of large-volume infusions as the neonatal PN containers. Three batches of each specification of 5% glucose injection were randomly selected, with 6 bottles measured per batch. After draining all the drug solution, the bottles were weighed, and the average value was set as the empty bottle weight of the infusion (Table 3).

**Table 3:** Empty Bottle Weight of PN Infusion Containers (g).

Drugs	Batch	Sample1	Sample2	Sample3	Sample4	Sample5	Sample6	Mean	RSD
5%Glucose 50mL	1	16.1	16.0	16.0	15.3	15.7	16.1	15.8	1.72%
	2	15.5	15.8	16.2	15.6	15.5	16.1		
	3	15.3	15.7	15.8	16.0	15.6	16.2		
5%Glucose 100mL	1	15.5	15.7	16.0	15.9	15.4	15.6	15.8	1.46%
	2	16.0	15.9	15.5	16.1	15.8	15.9		
	3	15.7	15.6	16.1	16.2	15.9	15.8		
5%Glucose 250mL	1	25.4	25.9	25.8	26.0	25.9	25.7	25.76	0.79%
	2	25.5	26.0	25.7	25.9	25.4	25.9		
	3	25.6	25.9	25.5	25.4	25.6	25.8		
5%Glucose 500mL	1	33.9	33.9	33.8	33.0	33.1	33.3	33.4	1.17%
	2	33.3	33.7	33.1	33.4	33.2	34.0		
	3	33.6	34.0	32.9	33.0	33.8	33.5		

### 2.7. Development of the intelligent weight verification management system

#### 2.7.1. Theoretical weight calculation.

Drug densities and empty bottle weights were maintained in the Hospital Information System (HIS) [10]. Through the informatization platform, automatically generate the following formula and calculate the theoretical weight value:


$$\begin{array}{c} W_{\text{theoretical}}=\;\sum_{i}\left(V_{i}\;p_{i}\right)+W_{\text{empty bottle}} \end{array}$$

Where *W* represents weight, *V*_*i*_ is the volume of each drug (mL), and $p_{i}$ is the density (g/mL).

#### 2.7.2. Actual weight measurement.

After weighing the ready-to-use infusions on a high-precision balance, the actual weight was recorded. The error rate was calculated as:


$$\begin{array}{c} E=\;\frac{W_{actual}\;-W_{theoretical}\;}{W_{theoretical}}\;\times \text{1}\text{0}\text{0}\% \end{array}$$

Where *E* represents the error rate; *E* > 0 indicates a positive deviation, and *E* < 0 indicates a negative deviation.

#### 2.7.3. Threshold setting and warning.

Foundation for Establishing the Preset Qualified Error Range (−1% to 5%):

① International standard foundation: ISO 7886–1:1993 and the parenteral nutrition safety specifications issued by JPEN both stipulate that the allowable range of error rate for parenteral nutrition preparation and weighing is ± 5% [11,12]. ② Clinical characteristics of neonates: Neonates have a significantly lower tolerance to dosage deviations than adults, therefore, the lower limit was tightened to −1% based on clinical risk assessment, which prevents false positive judgments caused by drug residual liquid and minor weighing errors, and effectively identifies the risk of negative deviations due to inadequate dosages. ③ Pilot experiment verification: A pilot experiment conducted in the early stage of this study on 50 bags of infusions verified that the threshold range of −1% to 5% can balance clinical safety and practical operational feasibility.

**Fig 1:**
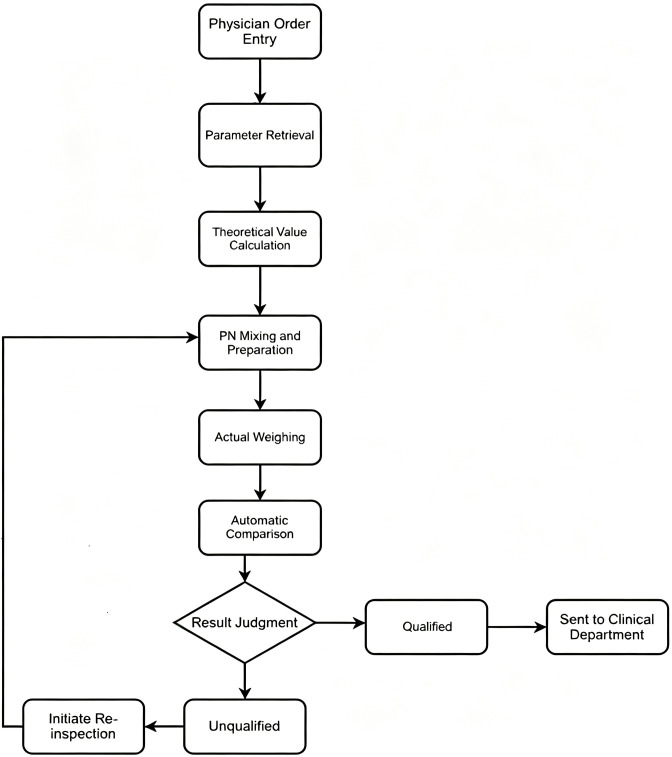
Workflow Chart of Intelligent Weight Verification Management System.

The HIS system automatically calculates and displays the threshold range of theoretical weight, and compares the actual weighing value with it. If it exceeds the range, the finished infusion is deemed unqualified and shall be reconstituted, then weighed again for judgment (Fig 1: Workflow chart of intelligent weight verification management system)

### 2.8. Validation indicators

Method Reliability:Precision (RSD), error trend (control chart analysis), and operator stability (interquartile range [IQR] of box plots) were employed to evaluate it. Comparison of Efficiency: The Residual Liquid Method and the Weighing Method were employed to verify each sample, and the verification time for each method were recorded and their efficiencies were compared [13,14].

### 2.9. Statistical analysis

The statistical data analysis was carried out using SPSS 26.0 software. The paired t-test was employed for inter-group comparison, and quantitative data were presented as mean ± standard deviation. The 3σ range was computed using the mean (μ) of all error rates as the center, the standard deviation (σ), and μ ± 3σ. Values that exceeded this range were considered outliers. A statistically significant difference was indicated by a P value less than 0.05.

### 2.10. Ethics approval and consent to participate

This research did not involve animal or human subjects, so relevant ethical guidelines were not mentioned.

## 3. Results

### 3.1. Method reliability validation

#### 3.1.1. Precision.

Under identical conditions, the same ready-to-use infusion was weighed twice repeatedly using 6 balances from the same manufacturer. Data analysis showed that the RSD values of infusion weights were all ≤ 1%, indicating excellent instrument stability (Table 4).

**Table 4:** Weighing precision of ready-to-use infusions.

Drugs	Mean(g)	SD	RSD
Ready-to-Use Infusion 1	89.1	0.12	0.13%
Ready-to-Use Infusion 2	271.5	0.10	0.02%
Ready-to-Use Infusion 3	538.1	0.09	0.04%

#### 3.1.2. Overall trend.

**Fig 2:**
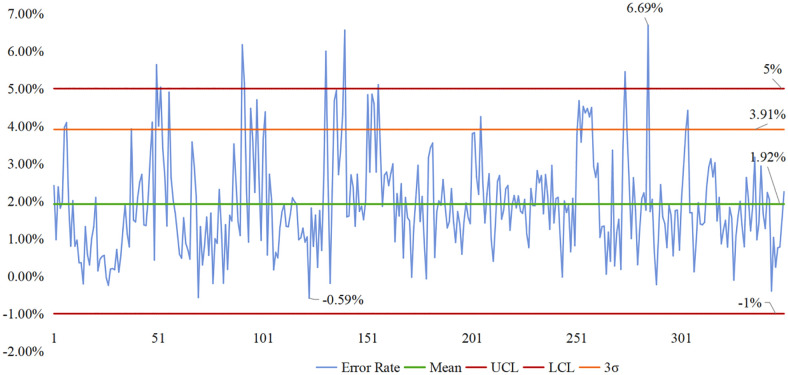
Control Trend of Overall Error Rate.

According to current international standards, the maximum threshold for the weighing method was set at 5%, and the minimum threshold was set at −1% based on practical conditions; values exceeding these thresholds were considered unqualified ready-to-use infusions. Statistical analysis of the weighing data showed that 97.43% of error rates fell within the threshold range, and 90.9% were within the 3σ range (Table 5, Fig 2: Control trend of overall error rate), indicating that the overall trend of error rates measured by the weighing method was within a controllable range and conformed to quality fluctuation patterns.

**Table 5:** Evaluation Indicators for Overall Error Rate.

Number of groups with positive dispensing error	Number of groups with negative dispensing error	Evaluation Indicators for Quality Error Rate (%) in Medication Admixture Process						SD	Number of values exceeding the threshold	Number of values exceeding 3σ
		Maximum	Median	Minimum	Upper threshold	Lower threshold	Mean			
336	14	6.69	1.72	−0.59	5	−1	1.92%	0.013	9	32

#### 3.1.3. Trend of error rate vs. fill volume variation.

**Fig 3:**
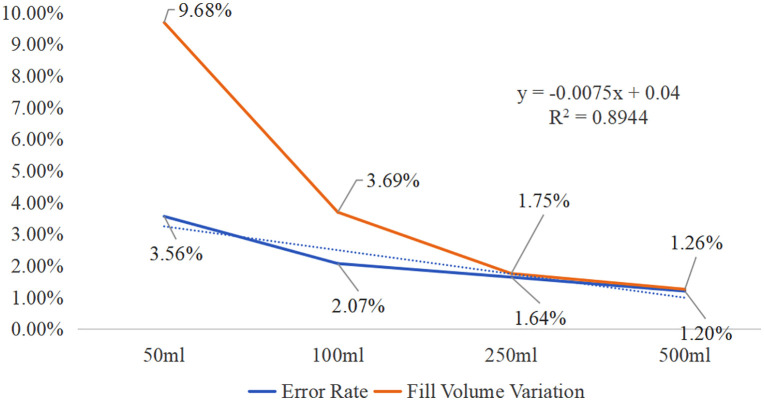
Correlation Between Error Rate and Fill Volume Variation.

Neonatal ready-to-use PN infusions use containers of corresponding specifications based on total liquid volume. To verify whether the error rate was correlated with fill volume variation, the PIVAS team measured the fill volume variation of 5% Glucose Injection in different specifications [15] and conducted comparative analysis with the error rates of corresponding infusions. The correlation coefficient k = −0.0075 indicated a negative correlation between the error rate and total liquid volume, consistent with the variation in fill volume of 5% Glucose Injection. Meanwhile, the linear regression coefficient R^2^ = 0.8944 showed a significant linear relationship between the error rate and total liquid volume (Fig 3: Correlation between error rate and fill volume variation). These results indicate that the error rate measured by the weighing method aligns with the pattern of fill volume variation, validating its applicability as a verification method for ready-to-use infusions.

#### 3.1.4. Dispenser operational stability.

**Fig 4:**
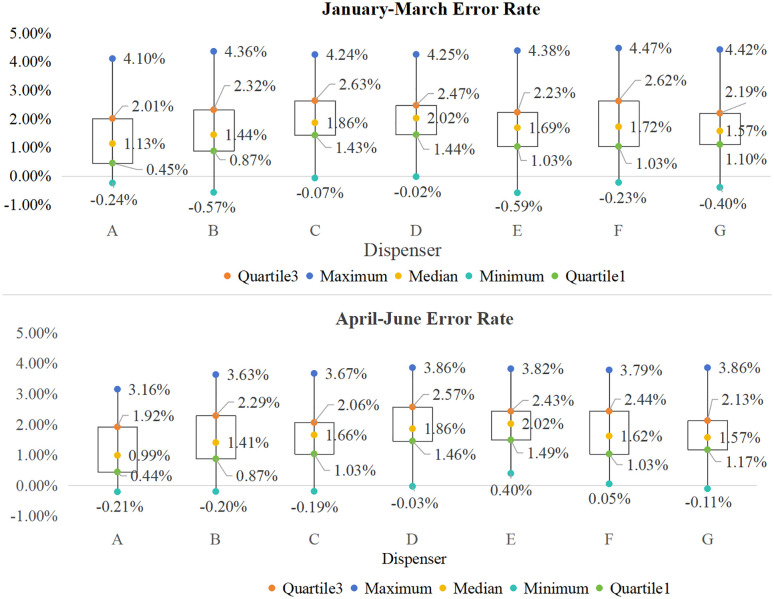
Comparison of Error Rates Among Different Dispensers.

Data from January to June were divided into two groups: January-March and April-June, with unqualified ready-to-use infusions (E ≥ 5%) excluded from both. The error rate data of each dispenser were statistically analyzed and box plots were plotted to visually reflect dispenser operational stability. The height of the box (IQR = Q3 - Q1) indicates data dispersion: a smaller IQR value signifies a more concentrated error rate distribution and greater dispenser stability. As shown in Fig 3, the IQR value changes among dispensers across different time periods were < 30%, indicating good operational stability. As shown in Fig 4: Comparison of error rates among different dispensers, the IQR variation of operators in different time periods was less than 30%, and there was no significant difference in the average error rates among junior, intermediate, and senior technicians (P > 0.05). The error rates of all technicians presented a slow downward trend from January to June without significant fluctuations, indicating that operator experience had a relatively minor impact on this system. The learning effect only brought a slight improvement in efficiency without changing the stability of the method, demonstrating good operational stability of the system.

### 3.2. Application effect

#### 3.2.1. Efficiency improvement.

Each ready-to-use infusion bag was verified successively using the residual liquid method and the weighing method, with verification times recorded. Data were analyzed via T-test using SPSS 26.0 (Table 6). Results showed that the weighing method reduced verification time to 15.4 minutes per bag, a 46.15% decrease compared to28.6 minutes per bag with the residual liquid method, with statistically significant difference (P < 0.05).

**Table 6:** Comparison of verification rates between residual liquid method and weighing method.

Method	Verification Rate (min/bag)
Residual Liquid Method	28.6(n = 350 bags)
Weighing Method	15.4(n = 350bags)^*^

Note: * indicates P < 0.05.

#### 3.2.2. Quality improvement.

The Weighing Method additionally detected six unqualified infusions exceeding the threshold, all with positive deviations. Among them, 4 bags had an excessive total liquid volume due to overfilling of large-volume injections; the other two bags were respectively caused by improper measurement of small volumes of 10% sodium chloride injection and calcium gluconate injection. Such deviations are difficult to identify through the residual liquid method..The Weighing Method significantly improves the level of infusion quality control and compensates for the Residual Liquid Method’s missed detection blind spot.

## 4. Discussion

The liver and kidney functions as well as the metabolic system of neonates, especially premature infants and very low birth weight infants, are immature. Even minor dose deviations may lead to severe adverse consequences such as electrolyte disorders, blood glucose fluctuations, and even organ damage [16]. Currently, clinical verification methods, such as the residual liquid and dual-operator verification methods, can meet the basic verification requirements. However, constrained by factors such as subjective judgment, visual errors, and work intensity, they pose a prominent risk of missed detections in the scenario of neonatal PN characterized by “small doses and multiple components.” The weight verification management system constructed in this study, through the full-chain design of “density quantification–information-based calculation–weight verification,” has realized the transformation of verification methods from “subjective qualitative” to “objective quantitative.” It provides a brand-new technical approach for the quality control of neonatal PN, and its core value and practical significance can be deeply discussed from the following dimensions.

### 4.1. Innovation of the technical system: resolving core technical bottlenecks in neonatal PN verification

#### 4.1.1. Accurate construction of a density database.

Among the commonly used drugs in neonatal PN, dose errors of small-volume and high-concentration drugs (e.g., 50% Glucose Injection and 10% Sodium Chloride Injection) have a significant impact on therapeutic effects. However, owing to the small sampling volume and the high difficulty in density determination, conventional methods struggle to achieve accurate quantification. In this study, two methods, “direct measurement with a densitometer” and “volume-mass conversion calculation,” were adopted for large-volume and small-volume drugs, respectively. Through the design of multi-batch and multi-repetition measurements, the relative standard deviation (RSD) of the obtained density data was ≤ 1%, which is far higher than the requirements for routine clinical detection, providing reliable basic parameters for the calculation of the theoretical weight. Particularly, for small-volume drugs, gradient sampling using syringes of different specifications (1 mL, 5 mL, 10 mL) effectively avoided systematic errors during micro-sampling. This design fills the gap in the domestic density database of PN drugs, specifically for neonates.

#### 4.1.2. Collaborative integration of information technology and weighing technology.

Traditional verification methods rely on manual calculations and visual judgments, resulting in low efficiency and high error rates. This study built an automated calculation module based on the Hospital Information System (HIS) and preset parameters, such as drug density and empty bottle weight, into the system. The theoretical weight and threshold range (−1% to 5%) were automatically generated through formulas, realizing the full-process automation of “medical order entry - parameter calling - theoretical value calculation - threshold early warning.” Meanwhile, an electronic balance with an accuracy of ±0.1 g was used for actual weight measurements, and objective quantitative evaluation was achieved through the error rate formula. This collaborative model of “information technology + precise weighing” minimizes manual intervention links, significantly improving the accuracy and efficiency of verification.

#### 4.1.3. Scientific basis for threshold setting.

International standards specify an allowable range of ±5% for PN preparation error rates; however, neonates have much lower tolerance for dose deviations than adults. Through a retrospective analysis of clinical data and risk assessment, this study tightened the lower limit of the threshold to −1%. This not only avoids false-positive determinations caused by drug residues and weighing errors but also effectively identifies the risk of insufficient doses caused by “negative deviations.” From the results, 97.43% of the error rates fell within the set threshold, and 90.9% were within the 3σ range, which conforms to the statistical laws of quality control. This indicates that the threshold setting balances “safety” and “practicality,” providing a scientific judgment standard for neonatal PN verification.

### 4.2. Multi-Dimensional verification of method reliability: Comprehensive coverage from laboratory precision to operator stability

#### 4.2.1. Dual guarantee of precision and trend controllability.

Method precision is a core indicator for evaluating the reliability of a verification system. In this study, through the design of “repeated measurement of the same ready-to-use infusion with multiple instruments,” the RSD of the obtained weight data was ≤ 0.13%, indicating that both instrument precision and method repeatability reached an extremely high level. Simultaneously, control chart analysis showed that the distribution of error rates presented an obvious normal trend, and the error rates of ready-to-use infusions with different volumes had a significant negative correlation with filling volume variations (R² = 0.8944). This result not only verifies the scientific validity of the method but also suggests that the system can adapt to the verification needs of PN with different specifications. In particular, it has an outstanding ability to identify errors in small-volume ready-to-use infusions (e.g., 50 mL), which is consistent with the clinical application scenario of neonatal PN.

#### 4.2.2. Clinical adaptability of operator stability.

The Pharmacy Intravenous Admixture Services (PIVAS) have high work intensity and frequent personnel turnover; therefore, the stability of the verification method is easily affected by the operator’s skill level and work status. By comparing the error rate data of seven compounding technicians in two periods (January-March and April-June), this study found that the variation in the interquartile range (IQR) values in different periods was < 30%. This indicates that the system has a low dependence on operators and can still maintain a stable verification effect even in cases of personnel rotation and work fatigue. This feature enables it to meet the large-scale and high-intensity work requirements of PIVAS, laying a foundation for clinical promotion.

### 4.3. Clinical application efficacy and popularization prospect in medical institutions

#### 4.3.1. Popularization prospect in medical institutions.

The intelligent weight verification and management system adopts a modular design with standardized interface protocols and a highly compatible, scalable underlying framework, enabling flexible secondary development and personalized functional customization.The system supports data interconnection with the Hospital Information System (HIS) and Laboratory Information System (LIS). In accordance with the actual operational workflows of the Pharmacy Intravenous Admixture Service (PIVAS), on-site interface adaptation, parameter configuration and system debugging can be efficiently implemented.This system provides robust technical support for clinical pharmacy management and standardized neonatal nutrition management in medical institutions, demonstrating promising clinical applicability and popularization potential.

#### 4.3.2. Clinical significance of incremental detection rate.

In the verification of 350 bags of ready-to-use infusions, the weighing method detected an additional six unqualified bags, with an incremental detection rate of 33.3%. Most of these missed detections were caused by issues such as “micro-dose deviations” and “uneven drug residue” which are difficult to identify via the residual liquid method. From the perspective of clinical risks, micro-deficiencies of components such as amino acids and electrolytes in neonatal PN may lead to adverse consequences such as growth retardation and electrolyte disorders. The application of the weighing method can effectively fill this blind spot of missed detection, adding a key line of defense for the safety of neonatal treatments.

#### 4.3.3. Cost-benefit analysis of verification efficiency.

One of the core obstacles to clinical promotion is the feasibility and cost-benefit of the method. The results of this study show that after adopting the combined weighing method, the verification time per bag of ready-to-use infusion only increased by 0.8 s, which was not significantly different from that of the residual liquid method (P > 0.05), and no additional complex equipment investment was required. From the cost-benefit perspective, this method significantly improves the detection rate of unqualified products with extremely low time cost and equipment investment, reduces adverse events and medical costs caused by dose errors, and thus has high clinical promotion value.

### 4.4. Limitations and future optimization directions

#### 4.4.1. Limitations and applicability of the single-center research design.

Due to its single-center design, this study includes certain limitations: ① The research objects were only neonatal parenteral nutrition infusions prepared in the PIVAS of our hospital, with relatively unified drug varieties, preparation procedures and operator abilities. Variations in PIVAS equipment conditions, operational specifications and drug varieties among hospitals must be taken into account when projecting the findings to other medical institutions; ② Glass bottles from various manufacturers and plastic flexible bags were not covered, and only 5% glucose injection was employed as the infusion container. The standardization and modularization of the weighing verification method developed in this study, however, allow other medical institutions to modify core indicators such as density parameters and empty container weight in accordance with their own circumstances, which provides a certain foundation for applicability. To further confirm the method’s universality, multi-center and large-sample studies can be conducted in the future to incorporate PIVAS data from hospitals of various levels in various regions.

#### 4.4.2. Expansion of container coverage.

Only empty bottles of 5% Glucose Injection were used as holding containers in this study. In the future, it is necessary to expand the coverage of container types (e.g., plastic bags and glass bottles of different specifications) and establish a more comprehensive parameter database.

#### 4.4.3. Correction of the impact of environmental factors.

Drug density is significantly affected by environmental factors, such as temperature and humidity. Although drug densities were measured at a room temperature of 20°C in this study, the actual compounding environment temperature in clinical practice may fluctuate (e.g., high temperatures in summer and low temperatures in winter), which is likely to cause deviations in density data. In the future, a temperature compensation module can be added to the HIS to automatically correct the density parameters according to the real-time environmental temperature, further improving the environmental adaptability of the system.

#### 4.4.4. Construction of full-process quality traceability.

The current system only covers the “post-compounding verification” link and does not involve the entire process of “medical order review - drug compounding - ready-to-use infusion administration”. In the future, weight verification data can be connected with the neonatal electronic medical record and PIVAS traceability system to realize the closed-loop management of “dose deviation-cause analysis-continuous improvement”. Simultaneously, it can provide objective data support for the traceability of adverse events.

### 4.5. Comparative analysis of the weighing method and spectroscopy

#### 4.5.1. Advantages and disadvantages of the weighing method.

Advantages: ① Simple operation: No complex sample pretreatment is required, and verification can be completed by direct weighing, making it appropriate for the high-workload clinical scenario of PIVAS; ② Low cost: All that is required is a high-precision electronic balance with low maintenance cost, which is suitable for promotion in primary medical institutions; ③ Fast and effective: The verification time for a single bag is only 15.4 minutes, which is significantly less than the spectroscopy detection time, enabling rapid verification of batch preparations; ④ Accurate quantification: The quantitative error rate for the total weight of infusions is less than 2%, which can satisfy the quality control requirements of neonatal parenteral nutrition.

Disadvantages: It must be implemented in conjunction with other techniques for component qualitative analysis given that it can only achieve quantitative verification of the overall weight of infusions and cannot detect the content deviation of individual components.

#### 4.5.2. Advantages and disadvantages of raman spectroscopy.

Advantages: It has a high detection specificity, can reliably identify a drug’s dose deviation or mismatch, and can perform both qualitative and quantitative analysis of individual components.

Disadvantages: ①Complicated operation: Professional technicians must do laborious sample pretreatment, which is challenging to adjust to the batch verification requirements of PIVAS; ② High cost: Raman spectroscopy instruments are expensive with high maintenance cost, which is difficult for primary medical institutions to afford; ③ Time-consuming detection: Rapid batch verification is impossible due to the lengthy detection time for a single sample; ④ Susceptible to matrix interference: Parenteral nutrition infusions contain complex components, which are prone to matrix interference and affect the accuracy of detection results.

In summary, the Weighing Method is more suitable as the routine batch quality verification method for neonatal parenteral nutrition infusions in PIVAS, while Raman spectroscopy can be used as a supplement for the cause analysis of unqualified infusions and the accurate detection of individual components.

### 4.6. Considerations on training, cost and scalability for system promotion on a wide scale

Training: Formulate a standardized operating manual and provide PIVAS operators with one to two days of specialized training covering system operation, density parameter maintenance, balance calibration and error analysis. Operators can only take up their posts after passing the practical operation assessment, with low training cost and short cycle.

Cost: The core equipment is a high-precision electronic balance with no additional consumable cost. The development of the Hospital Information System (HIS) module is an in-hospital information transformation without external payment, making the overall cost controllable and appropriate for medical institutions at all levels.

Scalability: The system adopts a modular design that makes it easy to connect to the HIS and Laboratory Information System (LIS) of various hospitals. It allows for the flexible addition and modification of drug density and container parameters, and it can be extended to the quality verification of pediatric and adult parenteral nutrition infusions, as well as other intravenous infusion preparations, with strong scalability.

## 5. Conclusions and prospects

The weight verification management system for neonatal PN ready-to-use infusions constructed in this study, through accurate density determination, information-based theoretical calculation, and objective weight verification, has realized the standardization and quantification of the verification method. This significantly improves the detection rate of unqualified products without increasing the clinical workload. This system not only fills the technical gap in the precise verification of neonatal PN but also provides a promotable standardized scheme for the quality control of clinical nutritional support.

In the future, with the improvement of the database, addition of the environmental correction module, and construction of the full-process traceability system, this system will play a more important role in fields such as neonatal intensive care and pediatric clinical nutrition, safeguarding the treatment safety of critically ill neonates.

## Abbreviations

PIVAS: Pharmacy intravenous admixture services
PN: Parenteral nutrition
HIS: Hospital information system
RSD: Relative standard deviation
NICU: Neonatal intensive care unit
